# P02.194. Newly developed functional medicine program in diabetes: impact on clinical and patient reported outcomes - functional medicine and quality of life

**DOI:** 10.1186/1472-6882-12-S1-P250

**Published:** 2012-06-12

**Authors:** D Franic, C Snapp, R DeBusk

**Affiliations:** 1 University of Georgia, Athens, USA; 2 Family Medicine Residency Program Tallahassee Memorial Healthcare, Tallahassee, USA

## Purpose

To determine the effectiveness and impact of a newly developed functional medicine program in diabetes patients using widely used quality of life measures, instruments frequently/increasingly used in functional medicine, in addition to traditional clinical outcomes.

## Methods

Adult clinic patients with diabetes were eligible to participate in a longitudinal comprehensive mindfulness-based therapeutic lifestyle change program involving weekly educational classes dedicated to teaching wellness and lifestyle changes. Patient outcome assessments were administered at baseline, 3- and 6- months, and included traditional clinical parameters and self-completed patient reported outcome measures: Five Facet Mindfulnesss Questionnaire (FFMQ) and Medical Symptom Questionnaire (MSQ); two generic quality of life measures, EuroQol’s EQ5D and Short Form 12 (SF-12); and the Summary Diabetes Self-Care Activities Questionnaire (SDSCA), a diabetes specific instrument.

## Results

Twenty-six patients with diabetes (96% type 2) participated in the study [mean age of 64.1 years (SEM=2.05), 70% Female). Patients’ weight, waist circumference and BMI decreased over 6 months (p<.05). FFMQ’s nonjudging on inner experience dimension improved over 6 months (p=.004). MSQ’s joint, weight, energy and emotion dimensions improved over 6 months (p<.05). EuroQol’s EQ5D-VAS showed patients’ overall quality of life improved (p<.05). SF-12’s role functioning and emotional health dimensions improved (p<.05, ES=.3 - .4). SDSCA showed significant improvements in diet, exercise, and glucose control (p<.05). By self-report at 6 months all diabetes patients participating reported improved quality of life.

## Conclusion

A functional medicine program piloted in a small group of patients with diabetes was effective. Patients showed significant improvements in traditional clinical parameters in addition to functional medicine metrics and widely used quality of life scales. This clinical trial is ongoing and expanded to include other conditions with the aim of integrating mindfulness based approaches to chronic disease into medical education at the Tallahassee Memorial HealthCare Family Medicine Residency Program.

